# Emerging and re-emerging pediatric viral diseases: a continuing global challenge

**DOI:** 10.1038/s41390-023-02878-7

**Published:** 2023-11-08

**Authors:** Seth A. Hoffman, Yvonne A. Maldonado

**Affiliations:** 1 Division of Infectious Diseases and Geographic Medicine, Department of Medicine, Stanford University School of Medicine, Stanford, CA USA; 2 Department of Pediatrics, Stanford University School of Medicine, Stanford, CA USA

## Abstract

**Abstract:**

The twenty-first century has been marked by a surge in viral epidemics and pandemics, highlighting the global health challenge posed by emerging and re-emerging pediatric viral diseases. This review article explores the complex dynamics contributing to this challenge, including climate change, globalization, socio-economic interconnectedness, geopolitical tensions, vaccine hesitancy, misinformation, and disparities in access to healthcare resources. Understanding the interactions between the environment, socioeconomics, and health is crucial for effectively addressing current and future outbreaks. This scoping review focuses on emerging and re-emerging viral infectious diseases, with an emphasis on pediatric vulnerability. It highlights the urgent need for prevention, preparedness, and response efforts, particularly in resource-limited communities disproportionately affected by climate change and spillover events. Adopting a One Health/Planetary Health approach, which integrates human, animal, and ecosystem health, can enhance equity and resilience in global communities.

**Impact:**

- We provide a scoping review of emerging and re-emerging viral threats to global pediatric populations
- This review provides an update on current pediatric viral threats in the context of the COVID-19 pandemic
- This review aims to sensitize clinicians, epidemiologists, public health practitioners, and policy stakeholders/decision-makers to the role these viral diseases have in persistent pediatric morbidity and mortality

In the first 2 decades of the twenty-first century, the world has witnessed an unprecedented number of viral epidemics and pandemics.^1–14^ This phenomenon coincides with the growing interconnectedness and interdependence of global society, facilitated by advancements in international trade, travel, and other forms of exchange. These events have been caused by both novel, emerging viral diseases and the resurgence of previously known, now re-emerging infectious diseases, which appear to exploit the impacts of climate change, the socio-economic connectedness of global society, geopolitical tensions, and social dynamics such as vaccine hesitancy and misinformation, as well as an imbalanced lack of access to vaccines, therapeutics, and other health interventions.^10,14–23^ There is a pressing need for ongoing global partnership and cooperation in surveillance, research, and preparedness measures to address current and future outbreaks, which require a multidisciplinary approach to tackle the complex interactions between the environment, socioeconomics, and health.^19,24^ Further, managing these events will also depend on consideration of low-probability, high-risk scenarios.

To develop a framework for preparedness planning for unpredictable events, the World Health Organization (WHO) introduced the concept of “Disease X” in 2018, well before the COVID-19 pandemic.^19,25^ “Disease X” represents a hypothetical pathogen, a ‘knowable unknown’, that could potentially trigger a future epidemic or pandemic. Since its introduction, “Disease X” has been included in the WHO’s list of emerging infectious diseases for research prioritization. The inclusion of “Disease X” highlights the need for preparedness measures that go beyond known pathogens and anticipate the emergence of new or unknown diseases. The WHO’s research and preparedness efforts for “Disease X” aim to develop effective interventions and response strategies that can be quickly deployed in the event of an outbreak of a new or unknown disease.^19,25^

The global pandemic caused by SARS-CoV-2 swept the world starting in late 2019 and resulted in the disease COVID-19.^11,12,26^ While preparation for “Disease X” resulted in calls to action and expert recommendations, chronically underfunded national and international public health institutions were slow to react and bogged down in multinational bureaucracy.^27,28^ Ultimately, as of April 2023 there have been over 640 million cases and nearly 8 million reported global deaths due to SARS-CoV-2 (the Institute of Health Metrics and Evaluation estimates total SARS-CoV-2-related deaths at over 18 million as of April 2022).^29^ According to the US Centers for Disease Control and Prevention (CDC), as of April 2023 there have been nearly 17 million cases and 2158 deaths due to SARS-CoV-2 in children and young people under the age of 18 in the United States.^30^ While transmission to children and young people are similar to adult transmission rates, differences in adult and pediatric mortality, with substantially lower mortality in children and young people, are thought to be mediated by age-associated immunological factors and the prevalence of comorbid conditions with age (increasing risk for hospitalization and death with age).^31,32^

The United States White House ended its national emergency concerning COVID-19 on May 11, 2023, after just over 3 years (enacted on March 13, 2020).^33^As our global community reflects on the response and aftermath of the COVID-19 pandemic, there is an urgent need for more effective preparedness and response strategies for emerging and re-emerging viral diseases. Children remain vulnerable to these diseases and the impact on their health and well-being can be severe.^26,34–38^ Understanding the trends in the emergence and re-emergence of viral diseases, as well as the current state of global efforts to prevent and control them, is critical for addressing this ongoing public health challenge.^23^ Moreover, the COVID-19 pandemic has revealed the detrimental impact of misinformation and disinformation on public health messaging, highlighting the complex interplay of sociocultural, psychological, and economic factors that shape effective and sustainable approaches to public health communication in the twenty-first century.^16,21–23,27,39,40^ Special attention must be paid to respiratory pathogens of pandemic potential, arboviral diseases whose vector ecology may change in the setting of climate change, and viral hemorrhagic fevers.^23^

In this scoping review, we showcase key emerging and re-emerging viral infectious diseases of significance, with particular attention to the vulnerability of pediatric patients. This review can inform clinicians, researchers, public health policymakers, stakeholders, and the lay public of the breadth by which to consider and stimulate action on prevention, preparedness, and response efforts. Emphasis must be placed on resource-limited communities that are most disenfranchised and most at-risk of the acute effects of climate change and spillover events, especially in emerging/re-emerging pathogen preparedness and response efforts. Focus on prevention efforts, which must include a One Health/Planetary Health framework, can provide more equity to all in our global community.^23^

## One Health/Planetary Health

One Health is defined as an integrated, unifying approach that aims to sustainably balance and optimize the health of people, animals and ecosystems^41,42^ and is related to Planetary Health, which seeks to address the interconnected challenges of climate change, biodiversity loss, and pollution, which all have negative impacts on human health—“Planetary Health is the health of human civilization and the state of the natural systems on which it depends.”^43,44^ Adapting a One Health/Planetary Health approach to combat emerging/re-emerging infectious diseases focuses attention on a key weakness of current prevention, preparedness, and response discourse: preventing spillover events, which bring zoonotic diseases in close proximity to humans and domesticated animals.^23^ All stakeholders must consider the effects of deforestation and land management, global animal husbandry and agricultural practices, and the global commercial wildlife trade.^23^

## Global action to turn polio into a disease of waning significance

Paralytic poliomyelitis is caused by the single-stranded positive-sense RNA poliovirus, a human-restricted enterovirus.^45^ Since the World Health Assembly unanimously voted to end global polio in 1988, global paralytic polio cases dropped from greater than 350,000 cases per year to only 30 cases at the close of 2022.^46–48^ This great human achievement can serve as a model for addressing other diseases which are candidates for either elimination or eradication, highlighting the principles of success to date due to the development of effective vaccines,^49,50^ global coordination and political will, and persistence in funding.^48^ However, despite the great progress that has been made in the fight against polio, political support for elimination efforts has waned. This is a major challenge that must be addressed if we are to achieve the goal of a polio-free world. There is a tremendous need for polio vaccine incentives, especially in the setting of decreasing polio vaccination rates, which have been linked to anti-vaccine groups and vaccine misinformation/disinformation, revisiting the global vaccination strategy as cases of vaccine-derived polio arise, as well as addressing underestimation of polio vaccine global production needs.^16,47,51,52^

## Emerging viral diseases

### SARS-CoV-2

SARS-CoV-2, the causative agent of COVID-19, is an enveloped, positive-sense, single-stranded RNA virus in the subgenera S*arbecovirus*, genus *Betacoronavirus*, family *Coronaviridae*.^11^ It is the cause of over 640 million cases and nearly 8 million reported global deaths since being first isolated in December 2019 in Wuhan, Hubei Province, China.^12,29,53^ Since the outset of the global COVID-19 pandemic, the impact of SARS-CoV-2 on pediatric populations has been variably described but appears to have low associated mortality when compared to adults, and yet high multi-factorial morbidity.^26,34–37,54,55^ Mechanisms and risk factors for the differential rates of infection and severe disease in children versus adults remain to be elucidated.^26,34,36,56–59^ One consequence of SARS-CoV-2 infection that appears to impact children at a higher rate is the subsequent development of multisystem inflammatory syndrome (MIS-C) as a severe complication, which manifests approximately 4–6 weeks after an initial asymptomatic or mild SARS-CoV-2 infection.^60,61^ The symptoms of MIS-C can vary, but can include persistent fever, chest pain, shortness of breath, rash, fatigue, abdominal pain, and diarrhea.^62^ Management of children diagnosed with MIS-C or children who develop hyperinflammation and respiratory difficulty associated with severe COVID-19 is centered around immunomodulatory treatments, antivirals such as remdesivir or in select cases Paxlovid (nirmatrelvir-ritonavir), and respiratory support.^26,63,64^ Children with chronic medical conditions appear to be at higher risk for severe COVID-19.^34,65,66^

Additional complications arising from acute SARS-CoV-2 infection include “post-COVID-19 condition” (PCC) or “long-COVID” or “post-acute sequelae of SARS-CoV-2 infection” (PASC) or “post-acute COVID-19 syndrome” (PACS), which refer to long-term symptoms and health effects that some individuals experience after recovery from acute SARS-CoV-2 infection,^67^ and can also occur in children.^35,38^ To date, there are no evidence-based treatments of PCC, and management is multidisciplinary including targeting specific symptom management. Specialty multidisciplinary pediatric PCC management clinics have been developed to support children with persistent symptoms. Of note, in one adult Veteran’s Affairs cohort study, treatment with the anti-viral Paxlovid (nirmatrelvir-ritonavir) resulted in reduced risk of PCC development, and in a phase 3 double-blind, randomized, placebo-controlled trial metformin was also associated with a reduced risk of PCC development.^68,69^

Non-pharmaceutical interventions, such as social distancing, hand hygiene and sanitizing practices, masking, and ventilation improvements have been shown to reduce COVID-19 transmission in children;^70,71^ and more broadly at the population level.^72–75^ However, any risk-mitigation intervention(s) must be taken in the context of minimizing impacts on pediatric mental health and well-being,^37,76^ as well as any social and economic impacts at local, national, and international levels that may require sustained individual effort to maintain their effectiveness. Improved indoor ventilation, air filtration, and air disinfection have been recognized as effective and sustainable alternatives to some of these measures.^77^

In the United States SARS-CoV-2 vaccines have been primarily the Pfizer/BioNTech Comirnaty BNT162b2 vaccine and the Moderna mRNA-1273 vaccine. These two vaccines have been shown to be safe and have varying degrees of effectiveness when given to children under the age of 18 for preventing infection, transmission, progression to hospitalization and severe COVID-19, death, MIS-C, and perhaps PCC.^78–87^ As of May 2023, the United States CDC recommends SARS-CoV-2 vaccination for all persons over the age of 6 months, while the WHO has deprioritized pediatric (6 months to 17 years) vaccination, communicating that globally the limited supply of vaccines should be prioritized for adults who are at higher risk of severe disease.^88,89^

SARS-CoV-2 remains a potent threat to human health and well-being, and more than serving as a model for future pandemics capable of disrupting the globe, it continues to kill over 5000 people per week—of which, the United States accounts for nearly 1/3 of cases (week of March 25, 2023).^90^ Further, between April 1, 2020—August 31, 2022, COVID-19 was a leading cause of death in children and young people aged 0–19 years.^91^

### Zika virus

Zika virus (ZIKV) is an enveloped, positive-sense, single-stranded RNA virus in the genus *Flavivirus*, family *Flaviviridae*; it is a mosquito-borne virus (arbovirus).^92^ In 2022 there were a reported >40,000 cases of ZIKV in the Americas.^93^ It was first discovered in Uganda in the late 1940s,^94,95^ but has spread globally due to the vector ecology of its preferred *Aedes aegypti* and *Aedes albopictus* mosquito vectors.^92,96^ After multiple outbreaks in the Pacific between 2007–2015, in which ZIKV presented much like dengue virus,^97–101^ an exanthematic disease began to impact northeastern Brazil in 2015—this likely reflected the introduction of ZIKV into a fully susceptible population.^8,102,103^ In late 2015, increased microcephaly cases were noted in the most ZIKV-affected areas of Brazil and the WHO subsequently declared a global public health emergency on February 1, 2016.^104,105^ Further case data has closely linked ZIKV maternal infection during the first two trimesters and subsequent development of microcephaly.^106–108^ The majority of acute cases of ZIKV suffer no complications, but the symptoms most commonly associated with disease in children include fever, rash, conjunctivitis, and arthralgia.^109–111^ Management of acute cases is supportive and there are no evidenced-based, approved therapies for use.^111^ Recommendations have been made on further subspecialty follow up of infants suspected to have congenital ZIKV infection and subsequent development of congenital ZIKV syndrome associated with microcephaly and other birth defects.^112^ The causative mechanism of microcephaly, and other birth defects associated with congenital ZIKV syndrome, is still not fully understood and serves as caution to the unanticipated effects of emerging infectious diseases.^113^ There are no approved vaccines or therapeutics specifically targeting ZIKV, but several are in the pipeline.^114^

### Nipah virus

Nipah virus (NiV) is an enveloped, negative sense, single-stranded RNA virus in the genus *Henipavirus*, family *Paramyxoviridae*.^115^ It was first isolated in 1999 after a Malaysian outbreak from 1998 to 1999 tied to pig farms, resulting in 283 cases and 109 deaths.^116–119^ Subsequent outbreaks of NiV in Bangladesh have been tied to the consumption of raw date palm sap that has been contaminated by *Pteropus* fruit bats (saliva, urine, feces).^120–122^ NiV can be transmitted person-to-person via droplet infection, but is particularly inefficient at using this mechanism of transmission as a driver of infection.^123,124^ Yet the possibility of developing more efficient person-to-person spread in a pathogen associated with mortality as high as >70%,^125^ has resulted in prioritizing NiV research due to its pandemic potential, particularly via the Coalition for Epidemic Preparedness Innovations (CEPI).^126^ Clinically, the disease course is rapid, severe, and associated with acute respiratory distress syndrome.^125^ In a review of 14 years of data on NiV transmission investigations in Bangladesh, individuals aged 14 years and younger were the most common primary case patients (37%), and within this age demographic, the estimated case fatality rate was nearly 80%.^127^ There are no approved vaccines or therapeutics specifically targeting NiV, but several are in the pipeline.^128^

### Mpox virus

Mpox virus (mpox; formerly Monkey Pox) is an enveloped, double-stranded DNA virus in the genus *Orthopoxvirus*, family *Poxviridae*.^129^ Mpox is a zoonotic disease (unknown animal reservoir),^129^ first isolated in 1958 subsequent to outbreaks of smallpox-like disease in cynomolgus monkeys kept in research facilities in Copenhagen, Denmark.^130^ During the course of smallpox surveillance and eradication campaigns, the first human case of mpox was detected in 1970 in a 9-month-old boy in Basankusu, Democratic Republic of Congo.^131^ Prior to 2017, outbreaks have been largely limited to the African continent and have not portrayed substantial risk of efficient person-to-person spread.^132–137^ Historical mpox case burden has been disproportionately reported in children.^136^ A 2003 outbreak of mpox in the United States resulted in over 72 confirmed or suspected cases in children and adults and was due to imported animals from Ghana (Gambian pouched rats that infected prairie dogs).^138^

In 2017–2018 a large outbreak of mpox occurred in Nigeria, primarily in male adults, with higher rates of person-to-person spread, and was hypothesized to involve sexual transmission and increased severity amongst people living with HIV.^139–141^ Starting in May 2022, the largest outbreak of mpox in history occurred, resulting in over 86,000 confirmed cases globally, mostly in the United States (>30,000 cases).^129,142^ The WHO declared the outbreak a public health emergency of international concern on July 23, 2022.^143^ The majority of cases in the 2022 mpox outbreak have occurred in men who have sex with men (MSM), >90% of cases across cohorts, with evidence of mucosal transmission through sexual contact as a possible key driver of transmission.^144–146^ While pediatric cases of mpox during the 2022 outbreak were rare (~40 US cases ≤15 years of age),^147^ prior data suggest an increased risk for severe disease in children.^133–135,148^ During the 2022 outbreak, the smallpox vaccine ACAM2000 (Pasteur Biologics Company) and the smallpox/mpox vaccine JYNNEOS (Bavarian Nordic) have been utilized for pre- and post-exposure prophylaxis,^149^ with a preliminary vaccine effectiveness of a single dose of subcutaneous JYNNEOS vaccine (full schedule includes 2 doses) of 86% reduction in risk for mpox in vaccinated individuals.^150^ The anti-viral TPOXX (tecovirimat; SIGA Technologies), an FDA-approved treatment for smallpox,^151^ was used for treatment of severe mpox under the FDA-regulated Expanded Access Investigational New Drug program, with encouraging but limited efficacy and safety data.^152–154^ Other drugs investigated, given pre-clinical effectiveness against mpox, include brincidofovir and cidofovir, but use of either drug is limited secondary to their side-effect profiles.^154,155^ Of note, a disproportionate fraction of mpox cases were experienced by people living with HIV,^144–146,156^ emphasizing the importance of education, communication, and prevention efforts in high-risk communities. Further, the case series identified an increased risk of progression to severe mpox disease and death in people living with advanced HIV/AIDS not on antiretroviral therapy,^157,158^ suggesting special attention to immunocompromised hosts. The global response to mpox has been effective in reducing the number of cases worldwide as of March 2023.^159^ However, this success is overshowed by issues in health inequity, especially as experienced by the African countries with long histories of mpox outbreaks, primarily affecting children, and wherein the disease is endemic. Despite the epidemiologic historical burden, these countries have not received equal access to mpox vaccines or antivirals.^149,160,161^

## Re-emerging infectious diseases

### Ebola virus

Ebola virus (EBOV) is an enveloped, negative sense, single-stranded RNA virus in the genus *Ebolavirus*, family *Filoviridae*, and the causative agent of Ebola virus disease (EVD).^162^ In 1976 simultaneous, but separate, viral hemorrhagic fever outbreaks in the Democratic Republic of the Congo (previously Zaire) and Sudan resulted in the isolation of EBOV.^163–167^ In these original outbreaks, the case fatality rate was 88% in Zaire and 53% in Sudan.^166,167^ Until 2013, most EBOV outbreaks occurred in Central Africa.^162^ The largest historical EBOV outbreak, declared an epidemic, occurred between late 2013 and early 2016 throughout West Africa (officially the 2014–2016 West African Ebola epidemic), spreading from Guinea to Liberia and Sierra Leone, and declared a WHO Public Health Emergency of International Concern on August 8, 2014.^168–170^ This outbreak resulted in more than 28,600 cases and 11,325 deaths, with an estimated adjusted case fatality ratio of nearly 83%.^171,172^ While children and young people represent a smaller proportion of overall cases, children under 5 years of age experience a shorter EVD incubation period and higher risk of death compared to older children and young people,^173,174^ emphasizing the importance of rapid assessment of young children in the context of future EBOV outbreaks.

Of note, a related filovirus—Marburg virus (MARV)—has resulted in outbreaks in Guinea, Ghana, Equatorial Guinea, and Tanzania between 2021–2023.^175^ Although data are limited, the impact of Marburg virus disease (MVD) on children and young persons is likely similar to that of EVD, highlighting the need for heightened surveillance.

### Measles virus

Measles virus (MV, also known as rubeola) is an enveloped, negative sense, single-stranded RNA virus in the genus *Morbillivirus*, family *Paramyxoviridae*.^176^ MV is transmitted via respiratory droplets and aerosols, is highly contagious, and lifelong immunity follows the survival of infection or vaccination.^176^ As there are no natural MV non-human reservoirs and there is a highly effective vaccine, MV elimination and eradication are possible and efforts to achieve these goals have been a global priority for decades. Prior to widespread measles vaccination, MV resulted in an estimated over 2.6 million global deaths per year, mostly among children under five years of age, dropping to over 760,000 global deaths in the year 2000 and down to just over 127,000 in 2021.^177^ Unfortunately, global MV cases and deaths have steadily increased since 2016, driven largely by a failure to vaccinate persons.^177,178^ Most of these deaths occur in children under the age of five.^177,178^ Children who are malnourished or have weakened immune systems, especially those living in low and middle-income countries (LMIC), are at the highest risk for severe illness and death.^176^ Additionally, complications of measles infection in children include blindness, deafness, and brain damage.^176^ The resurgence of measles in high-income country settings, and the failure to complete measles elimination in LMIC settings speak to the importance of persistent investment in global vaccination and surveillance, as well as the threat of vaccine hesitancy and vaccine misinformation/disinformation, which erode the public’s trust and imperil global vaccination efforts to prevent infection and disease.^16,179^

### Dengue virus

Dengue virus (DENV) is an enveloped, positive-sense, single-stranded RNA virus in the genus *Flavivirus*, family *Flaviviridae*, and the causative agent of dengue fever.^180,181^ It is a mosquito-borne virus (arbovirus) spread primarily by the *Aedes aegypti* mosquito (also, less commonly, via *Aedes albopictus*).^180,181^ There are four dengue serotypes (DENV-1, DENV-2, DENV-3 and DENV-4), and initial infection with one serotype followed by subsequent infection with another may result in severe dengue, associated with dengue hemorrhagic fever and dengue shock syndrome via antibody-dependent enhancement.^180–182^ Dengue is considered the most rapidly spreading mosquito-borne infectious disease in the world, with a recorded approximately 500,000 cases in the year 2000 increasing to over 5 million cases in 2019,^183^ while modeled estimates suggest true case burden is likely as much as 400 million cases per year in 2021.^183,184^ As is true for many arboviral diseases, dengue incidence is most common among children and young persons.^185^ Further, dengue infection is disproportionately more severe in children (including hemorrhagic disease and neurologic manifestations of disease), with severe consequences of maternal-fetal infection.^186–188^ Treatment of dengue is supportive, and there are no evidence-based, approved therapies or antivirals.^182^ There is one internationally licensed dengue vaccine with WHO prequalification, Dengvaxia produced by Sanofi Pasteur, which is indicated for adults, young people and children (from 6 to 45 years of age) with prior dengue virus infection confirmed by test; it is not indicated for travelers and is only approved in the United States for children (age 6–16 years) with laboratory-confirmed previous dengue infection and who live in endemic areas.^182,189,190^ Serologic testing for prior dengue infection is required prior to vaccination with Dengvaxia because persons without prior history of dengue infection are at-risk for severe dengue if they are infected with DENV after vaccination, similar to the risk of secondary serotype infection that causes antibody-dependent enhancement.^191^ There are several other vaccine candidates in the pre-clinical and clinical evaluation pipeline, some of which may not require pre-vaccination serologic testing.^192^

### Chikungunya virus

Chikungunya virus (CHIKV) is an enveloped, positive-sense, single-stranded RNA virus in the genus *Alphaviridae*, family *Togaviridae*, and the causative agent of chikungunya fever. It is a mosquito-borne virus (arbovirus) spread by *Aedes* mosquitoes (*aegypti, albopictus, africanus, furcifer-taylori, dalzeili*).^193^ First described during an outbreak of dengue-like illness between 1952-1953 in southern Tanzania, it has spread across the globe in a similar pattern as ZIKV subsequently did from Africa, to the Asia/Pacific region, and finally, to Europe and the Americas—all largely driven by the geographically and ecologically diverse habitats in which *Aedes* mosquitoes can thrive.^193–195^ Chikungunya fever is an acute febrile disease associated with severe arthralgia and arthritis, as well as headache and maculopapular rash—in comparison to DENV, CHIKV infection is more frequently associated with symptomatic disease.^193,196,197^ In children, cutaneous, neurologic, and hematologic manifestations are more broad and sometimes more common than in adults (although data are limited), including pigmentation, bullous rash, blistering, seizures, encephalopathy, thrombocytopenia, hemorrhage, and lymphopenia.^194,195,198–201^ There are no approved vaccines or therapeutics specifically targeting CHIKV, but several are in development.^201,202^

## A One Health/Planetary Health approach is key to global child health

The COVID-19 pandemic has highlighted the global community’s lack of preparedness for the next viral pandemic, given the political and socio-economic impacts of the current crisis. Unfortunately, we may not have time to prepare for impending new pandemics, as the global spread of highly pathogenic avian influenza H5N1 viruses has resulted in the culling or death of tens of millions of poultry and wild birds since 2022.^203,204^ The increasing mammalian spillover of H5N1, although still inefficient at human transmission, raises concerns about the ability of the virus to evolve as a more transmissible pathogen to mammals, including humans. The threat of another pathogen outbreak is compounded by the spread of misinformation and disinformation in a globally interconnected society. Moreover, the effects of climate change disproportionately impact under-served communities with scarce resources, resulting in widened gaps in health equity. Experts fear that climate change could increase the geographic spread of *Aedes aegypti*, causing a shift from long-standing infectious diseases of certain locations, such as malaria spread by *Anopheles* mosquitoes, to more arboviral diseases with limited vaccines and therapeutics.^205^ Preparing for low-probability, high-risk events, such as “Disease X”, will be of increasing importance. Addressing these issues requires international governmental partnerships that adopt a One Health/Planetary Health framework and ensure equitable distribution of resources, thereby preventing the disproportionate burden of emerging and re-emerging viral infectious diseases on all populations and especially children.

## Data Availability

Data sharing is not applicable to this article as no datasets were generated or analyzed during the current study.
